# Successful retrieval of a migrated stent in the pancreatic duct after endoscopic ultrasound-guided pancreaticogastrostomy with peroral pancreatoscopy

**DOI:** 10.1055/a-2239-4737

**Published:** 2024-02-15

**Authors:** Sho Takahashi, Ko Tomishima, Yusuke Takasaki, Akinori Suzuki, Shigeto Ishii, Toshio Fujisawa, Hiroyuki Isayama

**Affiliations:** 1 Department of Gastroenterology, Graduate School of Medicine, Juntendo University, Tokyo, Japan

Plastic stents are placed at anastomosis sites after endoscopic ultrasound-guided pancreaticogastrostomy (EUS-PGS) 1 2 . Straight plastic stents have better pushability than double-pigtail plastic stents but are more prone to migrate. We report a case of a straight plastic stent that migrated into the pancreatic duct (PD) and was successfully retrieved. It had been placed during EUS-PGS. The distal end was located at the tail side of the puncture point and was successfully retrieved using the guidewire technique by peroral pancreatoscopy (POPS).

A 68-year-old man underwent pancreaticoduodenectomy for distal bile duct cancer and repeated acute pancreatitis due to PD stricture and stones from alcoholic chronic pancreatitis. While attempting double-balloon endoscopic retrograde cholangiopancreatography for PD drainage, EUS-PGS was performed because an intestinal perforation had occurred at a previous institution.

Double-pigtail plastic stents and straight plastic stents were exchanged every 3 months after EUS-PGS. However, a straight plastic stent migrated into the PD ( Fig. 1 ). The distal end moved from the puncture point toward the pancreatic tail side ( Fig. 2 ). To retrieve it, a guidewire was inserted through the side hole of the flap on the jejunal end of the stent using POPS. Subsequently, the POPS scope was pushed to move the stent into the jejunum. Finally, the stent was completely pushed into the jejunum, and POPS was used to examine the distal end. The stent was successfully retrieved by grasping its distal end with a snare and pulling it back ( Video 1 ).

**Fig. 1:**
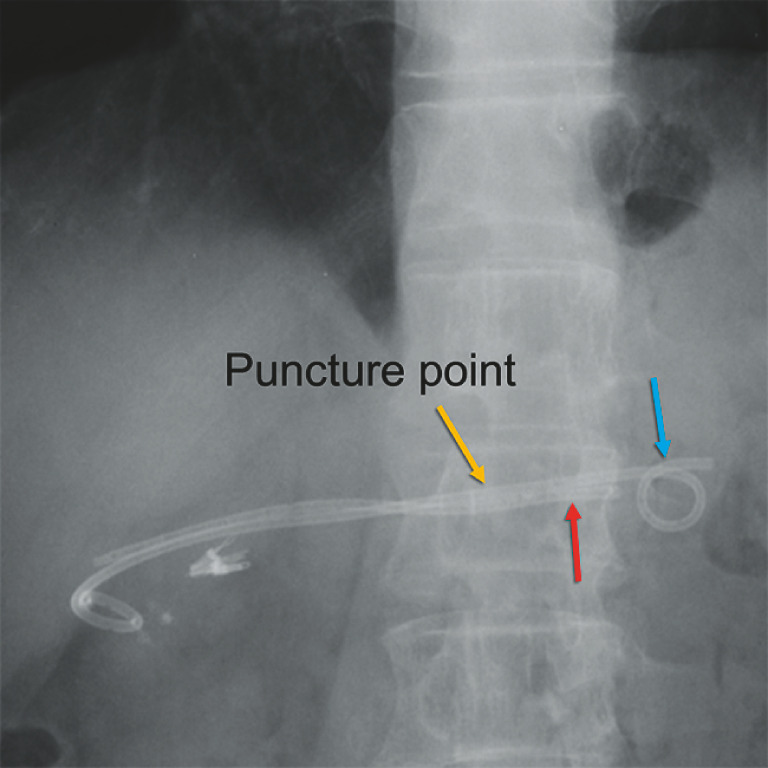
An X-ray image of this case. Yellow arrow: puncture point. Red arrow: gastric end of a straight plastic stent that had migrated to the pancreatic tail side. Blue arrow: gastric end of a double-pigtail plastic stent in the stomach.

**Fig. 2:**
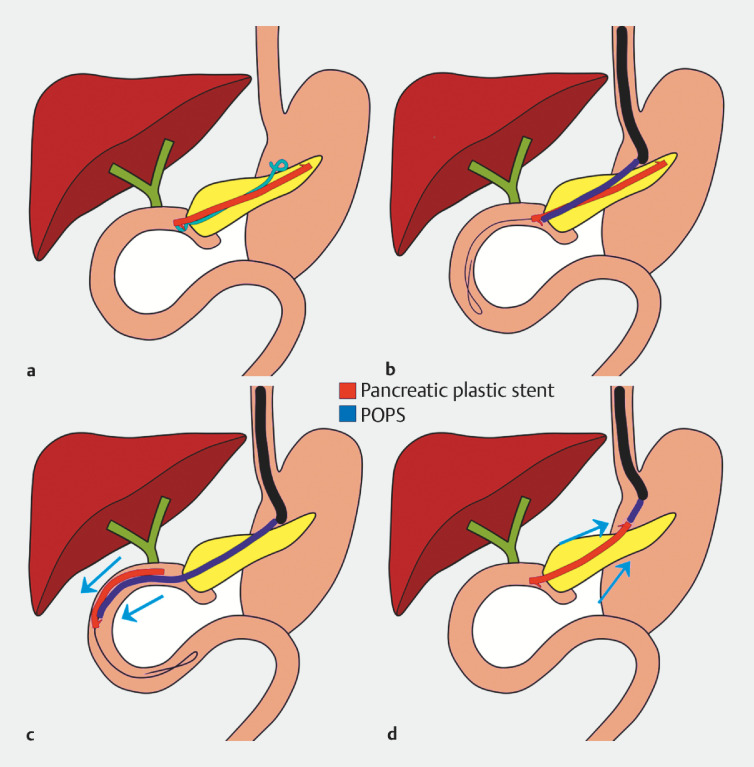
Schema of this case. **a** A straight plastic stent had migrated into the pancreatic duct. **b,c** The double-pigtail stent was removed. Then, using peroral pancreatoscopy and a guidewire, the straight plastic stent was pushed into the jejunum using the pancreatoscopy scope. **d** The stent was then successfully retrieved by grasping its distal end with a snare and pulling it back through the pancreaticogastrostomy.

A straight plastic stent placed during endoscopic ultrasound-guided pancreaticogastrostomy migrated into the pancreatic duct. The distal end of the plastic stent was located at the tail side of the puncture point and was successfully retrieved using the guidewire technique by peroral pancreatoscopy.Video 1

This may be the first case report describing a migrated straight plastic stent in the PD that was retrieved through the EUS-PGS route from the gastric side 3 4 5 . Retrieving a migrated plastic stent from the PD is challenging, even when the distal end of the stent is proximal to the puncture point. Using various devices and techniques with POPS led to successful removal in a difficult case of a migrated pancreatic plastic stent.

Endoscopy_UCTN_Code_TTT_1AR_2AI

## Notes

Endoscopy E-Videos https://eref.thieme.de/e-videos
*E-Videos* is an open access online section of the journal *Endoscopy* , reporting on interesting cases and new techniques in gastroenterological endoscopy. All papers include a high-quality video and are published with a Creative Commons CC-BY license. Endoscopy E-Videos qualify for HINARI discounts and waivers and eligibility is automatically checked during the submission process. We grant 100% waivers to articles whose corresponding authors are based in Group A countries and 50% waivers to those who are based in Group B countries as classified by Research4Life (see: https://www.research4life.org/access/eligibility/ ). This section has its own submission website at https://mc.manuscriptcentral.com/e-videos .
